# Washington Obstetrical and Genecological Society

**Published:** 1884-12

**Authors:** 


### Washington Obstetrical and Genecological Society.

At the annual meeting of the Washington Obstetrical and Gynecological Society, held October 7, 1884, Dr. Samuel C. Busey was re-elected President. The following officers were also elected for the ensuing year: Vice-Presidents, W. W. Johnston, M. D., and Joseph Taber Johnson, M. D.; Recording Secretary, C. H. A. Kleinschmidt, M. D.; Corresponding Secretary, Samuel S. Adams, M. D.; Treasurer, George Byrd Harrison, M. D. This young and vigorous society includes in its membership the best professional talent of the capital, and its proceedings are valuable contributions to the literature of obstetrics and gynecology.

---

Dr. Sullivan, in his address as President of the Canada Medical Association, spoke on the subject of homœopathy and the question of ethics. He also gave a history of the medical act of Ontario, which will be interesting to us all. After referring to the deplorable condition of affairs which existed prior to 1874, when the present law took effect, he said:

"The great evil which afflicted the profession was the ease with which doctors of all kinds were made, and it was primarily with a view to correcting it that overtures were made the homœopaths. In the midst of the dismal and unpropitious surroundings, it was seen that the profession must 'stoop to conquer,' and despite the opposition of the schoolmen and the forebodings of many, the 'regulars,' 'irregulars' and 'defectives' united, and agreed to erect a common standard on the subjects of anatomy, physiology, chemistry, botany, jurisprudence, and certain parts of surgery and midwifery, to which the medical act upon which they agreed obliged all, regardless of their views on therapeutics, who might subsequently enter the profession, to conform. The one portal was thus opened for all, and all other doors were closed. The result has been a condition of affairs in Ontario for which 'the United States sigh in vain, and for which England is struggling.' A higher grade of men and fewer of them are coming into the profession to take the places of those who, in the natural course of time, are obliged to drop out of the ranks, and (a result quite unlooked for) homœopathy, eclecticism and the various other isms have quite disappeared. When young men find it not less difficult to become homœopaths and eclectics than to become 'regulars,' they have almost invariably graduated as 'straight' physicians, without deviousness or irregularity. In Canada they have no code of ethics, the profession trusting to the amenities which should exist between gentlemen to protect each in his rights. Not to the Hindoo alone is loss of caste a penalty, and the lopping off of a member of a medical society for unprofessional conduct has a mighty effect on professional morals. In spite of the 'No code' which prevails across the border, the Canadian profession are not such an objectionable lot of men. Of course they consult with homœopaths, but barring this they compare very favorably, both as gentlemen and as scholars, with the profession of this country."

This calls to our minds the recent fight in this State, when those who desired to advance the standard of medical education were ignominiously defeated. Be it said to the honor of the homœopaths that they were on the side of right. Who, then, did defeat the proposed advance? The very men who cry, "Give us advance in medical education"; men who cry "quack" and "irregular," and are yet not only not trying to advance the standard, but openly fight this advance by raising objections to every conceivable plan which can be made. It is time for us to act. As it will be remembered, the subject was before the State society last year, and referred to a committee which will never report, or, if they do, will never report any bill which will at all strike at the evil which now exists. It would be well for the Erie county society again to take action and bring the matter before the legislature, and not wait for the State society, which, as we all know, is not in the least a representative body. When we have a bill such as Canada, such as Germany has, we will soon see, as it did in both of these, that all sectarianism will die. We sincerely hope that the medical schools in the State will soon realize their folly in opposing legislation, which, in the end, could only benefit them. If a medical faculty were established, it could only do good to medical schools of high standing, but it would prevent the poor schools sending their illiterate graduates into this State to compete, and that too often successfully, with men educated at our own schools, and who are well prepared for work. Every medical school in this State should have in their charter, as the Niagara medical school has, a provision for a three-years' course. Then, by establishing a medical faculty who would examine all who desire to practice in this State, our own graduates would have nothing to fear, and outside schools would be prevented from sending their graduates into this State to practice unless they were well educated. Let us hope and work that the standard of medical education in this State will be advanced this winter.

We learn that Dr. Frederick Peterson, of this city, has been appointed, after a severe competitive examination, to the position of Assistant Physician at the Poughkeepsie Insane Asylum. Dr. Peterson has demonstrated, through diligent study, his fitness for the highest professional honors. Such have been conferred upon him in this city, and he has ably met the highest expectations of his many friends. In the new field of labor upon which he is soon to enter, we are confident he will, with marked ability, meet its requirements and do honor to the profession and to the city which, in the past has fairly acknowledged his intellectual power and his professional attainments, and from which he bears an enviable record for scientific research and the best wishes of the medical fraternity.

---

The Committee on Organization of the next International Medical Congress, which is to be held in Washington, D. C., in 1887, has been constituted, as follows: Drs. Austin Flint, Sr., New York; I. Minis Hayes, Philadelphia; Lewis A. Sayre, New York; Christopher Johnson, Baltimore; George J. Englemann, St. Louis; J. S. Brown, U. S. Navy; J. S. Billings, U. S. Army.

---

Dr. Lawson Tait has returned to his home across the waters, and Dr. W. S. Playfair, author of an excellent text-book on midwifery, is now visiting America. Dr. Emmet, of New York, gave him a reception on Sept. 26th, memorable for the unusually large number of representative men who were gathered together from that city and vicinity.

---

Dr. Thomas R. French, of Brooklyn, N. Y., photographs the larynx, and obtains pictures of great clearness. The process can be used, it is claimed, with untrained patients. It is, as the *N. Y. Medical Journal* truly says, "a distinct advance in the demonstration and recording of laryngeal affections."

THE Third International Otological Congress was held at Basel, Switzerland, from September 1st to 5th. The work of the Congress was of an original and interesting character.

